# Propolis Ethanol Extract Stimulates Cytokine and Chemokine Production through NF-*κ*B Activation in C2C12 Myoblasts

**DOI:** 10.1155/2015/349751

**Published:** 2015-10-29

**Authors:** Kohei Washio, Mao Kobayashi, Natsuko Saito, Misato Amagasa, Hiroshi Kitamura

**Affiliations:** Laboratory for Veterinary Physiology, Department of Veterinary Medicine, School of Veterinary Medicine, Rakuno Gakuen University, 582 Bunkyodai-Midorimachi, Ebetsu 069-8501, Japan

## Abstract

Myoblast activation is a triggering event for muscle remodeling. We assessed the stimulatory effects of propolis, a beehive product, on myoblasts. After an 8 h treatment with 100 *μ*g/mL of Brazilian propolis ethanol extract, expression of various chemokines, including CCL-2 and CCL-5, and cytokines, such as IL-6, increased. This propolis-induced cytokine production appears to depend on NF-*κ*B activation, because the IKK inhibitor BMS-345541 repressed mRNA levels of CCL-2 by ~66%, CCL-5 by ~81%, and IL-6 by ~69% after propolis treatment. Supernatant from propolis-conditioned C2C12 cells upregulated RAW264 macrophage migration. The supernatant also stimulated RAW264 cells to produce angiogenic factors, including VEGF-A and MMP-12. Brazilian green propolis therefore causes myoblasts to secrete cytokines and chemokines, which might contribute to tissue remodeling of skeletal muscle.

## 1. Introduction

Myoblasts are progenitor cells for myocytes. Although these cells are quiescent in intact muscle, they start to proliferate under stress conditions such as exercise or tissue damage [1, 2]. Myoblasts and myocytes also produce the chemokines that attract monocytes to skeletal muscle [3]. After infiltration, monocytes undergo differentiation into macrophages and participate in tissue repair and remodeling. Macrophages elicit angiogenesis through the secretion of angiogenic factors such as VEGF and FGF-2 [3, 4].

Propolis is a beehive product that is rich in phenolic compounds. Numerous studies have demonstrated that propolis ethanol extract (PEE) exerts various biological effects, including tumoricidal, immunomodulatory, and antidiabetic effects [5–8]. In addition, PEE produced from Brazilian green propolis and its major chemical constituents, namely, artepillin C and coumaric acid, promote glucose uptake in skeletal muscle. This suggests that PEE has the potential to modulate the basic cellular function of mature myocytes [9]. Despite these pioneering studies, there is little information about the effects of propolis on myoblasts.

In this study, we attempt to clarify the modulatory roles of Brazilian propolis in myoblasts, focusing particularly on the secretion of cytokines and chemokines. We found that PEE induced expression of IL-6, LIF, CCL-2, CCL-5, and CXCL-10 in mouse myoblast C2C12 cells through NF-*κ*B activation. Moreover, propolis-conditioned myoblasts modulated macrophage migration and angiogenic factor expression, potentially contributing to the tissue remodeling of skeletal muscle.

## 2. Materials and Methods

### 2.1. Cells and Reagents

C2C12 cells and RAW264 cells were obtained from the RIKEN BioResource Center (Tsukuba, Japan) and maintained under standard conditions. PEE from Brazilian green propolis was supplied by the Yamada Bee Farm (Kagamino, Japan). Preparation of PEE was conducted as follows: Brazilian green propolis was homogenized in a 10-fold volume of ethanol and agitated at room temperature for 12 h. Subsequently, the solution was filtrated and evaporated until the solid content of the solution was 55% by weight. Total solid content mass was determined after evaporation* in vacuo* and further drying in an oven. Chemical composition of the propolis ethanol extract is shown in Table 1. When we added PEE to the C2C12 cells, we diluted 55% stock solution with dimethyl sulfoxide (DMSO) to 10% and then further diluted it 1,000-fold with serum-containing medium. The vehicle control group was treated with a solution containing 0.008% ethanol and 0.08% DMSO. I*κ*B kinase (IKK) inhibitor BMS-345541 was purchased from Calbiochem (Billerica, MA).

### 2.2. Cell Number Analysis

The number of viable C2C12 cells was measured using an XTT cytotoxicity assay kit (Roche, Basel, Swiss) according to the manufacturer's manual.

### 2.3. Quantitative Reverse Transcription-Polymerase Chain Reaction (qRT-PCR) Analysis

Total RNA, which was extracted with TRIzol reagent (Thermo Fisher, Waltham, MA), was subjected to qRT-PCR analysis as described previously [10]. The following primer pairs were used: 5′-AGGTCCCTGTCATGCTTCTG-3′ and 5′-TCATTGGGATCATCTTGCTG-3′ for CCL-2; 5′-CAGCACTTCACCCATCAGTT-3′ and 5′-GGCGCAGTTTATGTTGTCTG-3′ for cyclooxygenase 2 (COX2); 5′-AAGTGCTGCCGTCATTTTCT-3′ and 5′-CCTATGGCCCTCATTCTCAC-3′ for CXCL-10; 5′-AGCGGCTCTACTGCAAGAAC-3′ and 5′-AGCGGCTCTACTGCAAGAAC-3′ for FGF-2; 5′-GCTGAAAGCTCTCCACCTCA-3′ and 5′-AGGCCACAGGTATTTTGTCG-3′ for IL-1*β*; 5′-GTTCTCTGGGAAATCGTGGA-3′ and 5′-TTCTGCAAGTGCATCATCGT-3′ for IL-6; 5′-GGCAACCTCATGAACCAGAT-3′ and 5′-TAGGCGCACATAGCTTTTCC-3′ for LIF; 5′-AGTGGCAGGTAGAGCAGGAA-3′ and 5′-TTGCAAGGACATACGAGAGC-3′ for molecules possessing ankyrin repeats induced by lipopolysaccharide (MAIL)/I*κ*B*ζ*; 5′-GCTGTCACAACAGTGGGAGA-3′ and 5′-GAAGTAATGTTGGTGGCTGGA-3′ for MMP-12; 5′-CCACCACGCTCTTCTGTCTA-3′ and 5′-AGGGTCTGGGCCATAGAACT-3′ for TNF-*α*; 5′-GGAGAGCAGAAGTCCCATGA-3′ and 5′-ACTCCAGGGCTTCATCGTTA-3′ for VEGF-A. The predesigned dual-labeled probe and primer sets for CCL-5, hypoxanthine-guanine phosphoribosyltransferase-1 (HPRT-1), and 18S ribosomal RNA were purchased from Sigma-Aldrich Japan (Ishikari, Japan).

### 2.4. Enzyme-Linked Immunosorbent Assay (ELISA)

Culture medium from the C2C12 cells was collected and stored at −80°C until usage. The IL-6 content of this medium was measured using a Ready-SET-Go! ELISA kit (eBioscience, San Diego, CA) according to the user's manual.

### 2.5. Western Blot Analysis

Nuclear and cytoplasmic proteins were prepared with a nuclear extraction kit (Active Motif, Carlsbad, CA). SDS-PAGE and subsequent western blotting were performed as described previously [11]. Anti-p65, anti-p50, anti-I*κ*B*α*, anti-cRel, and anti-H3 histone antibodies were purchased from Cell Signaling Technology (Danvers, MA). Anti-GAPDH antibodies were purchased from Santa Cruz Biotechnology (Dallas, TX).

### 2.6. Cell Migration Test

The cell migration test was performed using a kit purchased from Platypus Technology (Fitchburg, WI). Briefly, supernatants from C2C12 cells stimulated for 12 h with PEE (100 *μ*g/mL) or vehicle were diluted and added to RAW264 cells in a kit-supplied plate. After staining with Hoechst 33342 (Thermo Fisher), cell migration was monitored using a laser-scanning confocal microscope ECLIPSE Ti (Nikon, Tokyo, Japan), 18 h after adding the supernatants. The fluorescence intensity was digitalized using the NIS-Elements Advanced Research software (Nikon) and further analyzed with ImageJ [12].

### 2.7. Statistical Analysis

Data were analyzed using Student's *t*-test or one-way analysis of variance (ANOVA), followed by Bonferroni's* post hoc* test, using KaleidaGraph software (Synergy Software, Reading, PA).

## 3. Results

### 3.1. PEE Induces the Production of Cytokines and Chemokines in C2C12 Myoblasts

**Figure 1:**
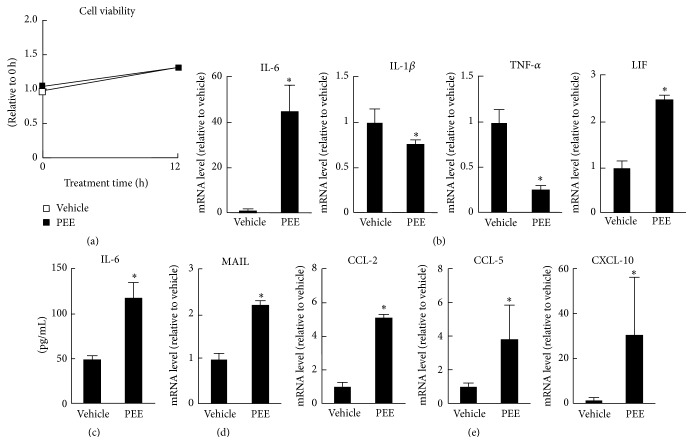
Effects of propolis ethanol extract (PEE) on mRNA expression of cytokines and chemokines in C2C12 myoblasts. C2C12 cells were treated with PEE (100 *μ*g/mL) from Brazilian propolis for 12 h (a, c) or 8 h (b, d, and e). (a) Cell number before and after PEE treatment. Data are represented as means ± SD of four samples. qRT-PCR analysis of (b) cytokines, (d) MAIL, and (e) chemokines in C2C12 cells treated with PEE or vehicle. Data normalized to HPRT-1 mRNA (means ± SD of four samples). (c) Accumulation of IL-6 in culture medium from C2C12 cells after treatment with PEE (means ± SD of eight samples). ^*∗*^ *P* < 0.05 versus vehicle-treated control cells.

First, we tested a dose of 100 *μ*g/mL PEE for toxicity to C2C12 cells. This dose did not affect cell viability 12 h after application (Figure 1(a)), so henceforward that was the dose used.

Under stressful situations, myocytes secrete cytokines, which cause inflammation in skeletal muscle [13]. To investigate whether PEE affects cytokine production in myoblasts, we measured the mRNA levels of various cytokines 8 h after PEE application. IL-6 mRNA expression increased more than 40-fold in C2C12 myoblasts (Figure 1(b)). In addition, the IL-6 content in the cells' culture medium 12 h after application was significantly higher in propolis-treated cells than in vehicle-treated ones (Figure 1(c)). In contrast, IL-1*β* and TNF-*α* decreased following PEE treatment. PEE also failed to induce IL-1*β* and TNF-*α* at 4 h (data not shown). The stimulatory effects of PEE thus appear to be selective for certain inflammatory cytokines only.

We next assessed MAIL/I*κ*B*ζ* expression in myoblasts, because MAIL promotes IL-6 induction in stromal cells in a relatively selective manner [14]. As expected, MAIL transcripts more than doubled in the PEE-treated C2C12 cells compared to the vehicle-treated cells (Figure 1(d)). Because MAIL has also been reported to potentiate expression of CCL-2 and LIF [15], we also measured the levels of these transcripts in the cells following PEE treatment. Both types of transcripts were significantly upregulated; CCL-2 levels were approximately 5.2 times higher and LIF about 2.5 times higher (Figures 1(b) and 1(e)).

In addition to CCL-2, we proposed that PEE might stimulate the induction of other chemokines in C2C12 cells. Indeed, both CCL-5 and CXCL-10 transcript levels increased by about 3.9 times and 31 times, respectively, relative to vehicle treatment (Figure 1(e)). PEE therefore induces the production of several chemokines in myoblasts.

### 3.2. NF-*κ*B Is Responsible for PEE-Induced Cytokine and Chemokine Production in C2C12 Myoblasts

**Figure 2:**
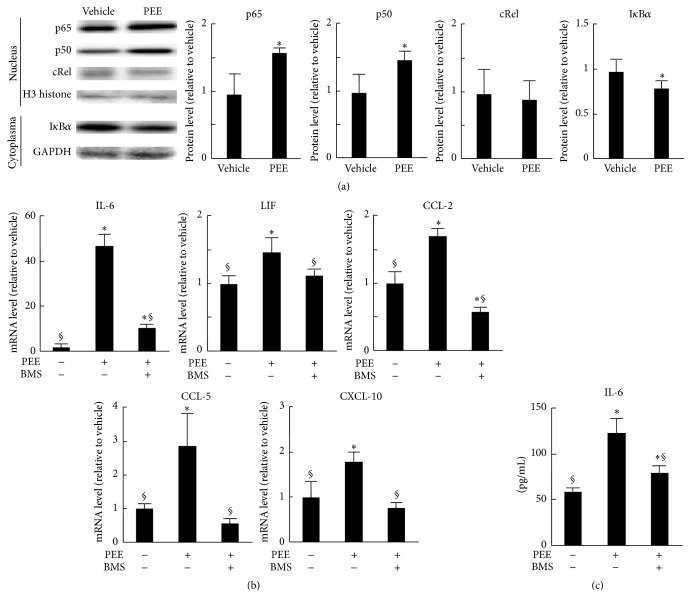
Role of NF-*κ*B p50 and p65 in propolis-elicited cytokine induction in C2C12 cells. (a) Western blotting analysis of nuclear p65, p50, and cRel and cytoplasmic I*κ*B*α* from cells treated with 100 *μ*g/mL PEE or vehicle for 3 h. H3 histone and GAPDH from nuclear and cytoplasmic protein, respectively, were used as loading controls. Left: representative blot images from three independent experiments. Right: quantitative data for band intensities. Data normalized to H3 histone (means ± SD of three samples). ^*∗*^ *P* < 0.05 versus vehicle-treated control cells. (b, c) Effects of 1 *μ*M of an IKK inhibitor BMS-345541 on PEE-elicited cytokine induction. (b) Results of qRT-PCR analysis after treatment with PEE for 8 h. Data normalized to HPRT-1 mRNA (means ± SD of five samples). (c) Accumulation of IL-6 in culture medium 12 h after PEE application (means ± SD of eight samples). ^*∗*^ *P* < 0.05 and ^§^ *P* < 0.05 versus vehicle-treated control cells and PEE-treated, BMS-345541-untreated cells, respectively.

Induction of IL-6, CCL-2, and CXCL-10 is highly dependent on NF-*κ*B in some cell types [16, 17]. We therefore monitored NF-*κ*B activation in the PEE-treated C2C12 cells. Western blotting showed that p65 and p50 NF-*κ*B components accumulated in the cells' nuclei 3 h after PEE treatment, while cytoplasmic I*κ*B*α* decreased significantly (Figure 2(a)). In contrast, nuclear cRel levels remained constant after PEE treatment. PEE therefore activates the NF-*κ*B proteins p50 and p65 in C2C12 cells.

We next assessed the involvement of NF-*κ*B in PEE-elicited cytokine and chemokine induction. To achieve this, we treated C2C12 cells with 1 *μ*M of the IKK inhibitor BMS-345541 1 h before PEE treatment. This treatment significantly decreased PEE-elicited induction of transcripts for IL-6, LIF, CCL-2, CCL-5, and CXCL-10 to 31.2%, 76.2%, 34.0%, 19.1%, and 42.2%, respectively (Figure 2(b)). It also attenuated the accumulation of IL-6 in the culture medium after PEE treatment (Figure 2(c)). NF-*κ*B therefore plays an important role in the induction of cytokine and chemokine production in C2C12 cells following PEE treatment.

### 3.3. PEE-Conditioned C2C12 Myoblasts Promote Macrophage Migration

**Figure 3:**
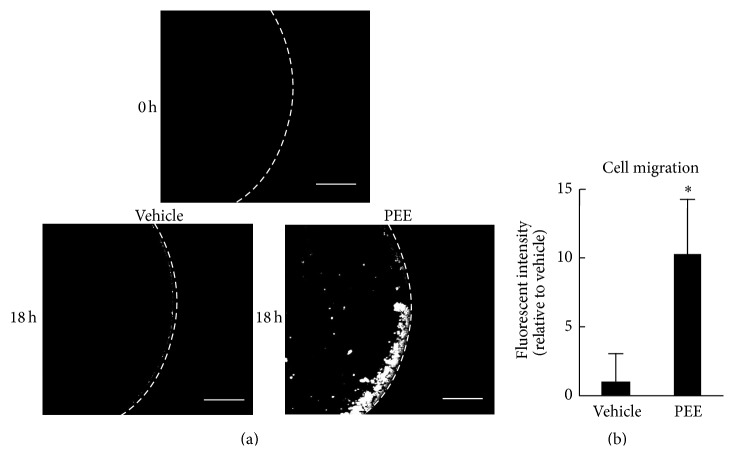
Effect of supernatants from PEE-conditioned C2C12 cells on macrophage migration. (a) Representative microscopic images of the RAW264 macrophages treated with supernatant from C2C12 cells treated with PEE or vehicle for 8 h. Scale bars represent 200 *μ*m. (b) Quantification of macrophage migration based on the wells' fluorescent intensity (means ± SD of four wells). ^*∗*^ *P* < 0.05 versus supernatant from vehicle-conditioned control cells.

CCL-2 is thought to be an important chemokine for introducing monocytes/macrophages to lesions. We therefore hypothesized that PEE-conditioned myoblasts would be able to accelerate macrophage migration. To investigate this, we measured the migration activity of RAW264 macrophages after application of supernatants from PEE- or vehicle-conditioned C2C12 cells. The PEE-conditioned supernatant caused RAW264 cell migration to increase to 10.3 times that of the vehicle-conditioned cells 18 h after application (*P* < 0.01; Figures 3(a) and 3(b)). PEE-stimulated myoblasts therefore have the potential to promote macrophage migration.

### 3.4. PEE-Conditioned C2C12 Myoblasts Stimulate Macrophages to Express Angiogenic Factors

**Figure 4:**
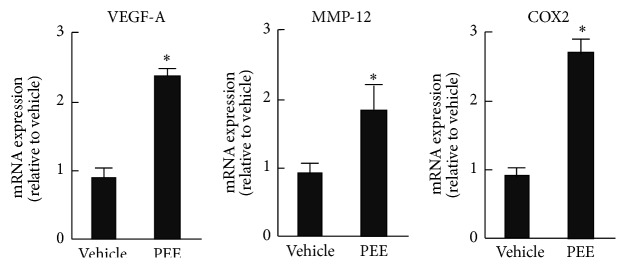
Effect of supernatant from PEE-conditioned C2C12 cells on mRNA expression of angiogenic factors in macrophages. Results of qRT-PCR analysis of RAW264 cells treated for 12 h with supernatant from C2C12 cells conditioned with either 100 *μ*g/mL PEE or vehicle for 8 h. Data normalized to 18S rRNA (means ± SD of four samples). ^*∗*^ *P* < 0.05 versus supernatant from vehicle-conditioned control cells.

After recruitment, macrophages mainly contribute to tissue remodeling and repair, and during tissue remodeling they elicit angiogenesis. We therefore tested whether PEE-stimulated cells potentiate the expression of angiogenic factors in macrophages. The mRNA expression of a major angiogenic factor, VEGF-A, was much greater in RAW264 cells treated with supernatant from PEE-conditioned C2C12 cells than in those treated with supernatant from vehicle-conditioned cells (Figure 4). This treatment had a similar effect on mRNA expression of MMP-12 and COX2, both of which contribute to angiogenesis [18], but not on FGF-2 transcript levels. These results indicate that PEE-stimulated myoblasts direct macrophages to induce production of several angiogenic factors that participate in tissue remodeling of skeletal muscle.

## 4. Discussion

In this study, we have demonstrated that PEE from Brazilian green propolis induces the production of cytokines and chemokines in C2C12 myoblasts. Induction of these humoral factors is likely to depend on NF-*κ*B p50 and p65 activation, because an IKK inhibitor clearly repressed PEE-elicited induction of cytokines and chemokines. Interestingly, the induction effects of PEE were selective for certain transcripts; transcripts for IL-6, LIF, CCL-2, CCL-5, and CXCL-10 increased strongly following PEE treatment, while those for IL-1*β* and TNF-*α* were unaffected after 4 h and had decreased after 8 h. The molecular mechanisms that underlie the induction of cytokine and chemokine production remain elusive. One possibility is that MAIL is involved in this induction, because MAIL modulates chromatin, which can lead to the selective induction of IL-6, LIF, and CCL-2 [14, 15]. Our data support this hypothesis, since they show that PEE strongly induced MAIL expression in C2C12 cells, in parallel with the aforementioned cytokine and chemokine induction.

Some reports about the effects of propolis and its chemical components on cytokine expression are subject to much debate [19, 20]. Geographical difference might be one of the main reasons for the observed variations in the anti- or proinflammatory effects of propolis. Poplar-type propolis contains caffeic acid phenethyl ester as a major bioactive component, whereas a major component of Brazilian green propolis is artepillin C [20, 21]. Since these chemical components have different inflammatory effects [22], they might result in different cytokine responses. Differences in cell types might be another determinant. Expression levels of intracellular signaling molecules vary between cell types, so the expressional control of cytokines also varies according to cell type. For example, expression of MAIL was relatively high in the myoblasts compared to the other types of cells, including monocytes, when intact (data not shown). Nevertheless, further studies are required to investigate the molecular mechanisms underlying the positive and negative effects of PEE on cytokine production in several cell types.

PEE from Brazilian propolis contains a variety of bioactive compounds, including artepillin C and chrysin, which have been reported to repress NF-*κ*B activation in various cell types [23, 24]. Our current data are therefore surprising, since they show that PEE activates NF-*κ*B in myoblasts under nontoxic conditions. This suggests that myoblasts may have a unique intracellular molecule(s) that sensitizes certain PEE-constituent chemicals and subsequently activates NF-*κ*B signaling.

PEE induced several chemokines in the C2C12 myoblasts; it therefore has the potential to recruit leukocytes to skeletal muscle. Of the chemokines, CCL-2 is considered to be a pivotal chemoattractant for monocyte/macrophage lineages [25]. It has recently been proposed that M2 macrophages are responsible for remodeling in many tissues, including skeletal muscle [26]. PEE-treated myoblasts might therefore repair skeletal muscle through the recruitment of M2 macrophages. This might account for the effectiveness of propolis for treating eccentric exercise-induced muscle injury.

Treatment with supernatant from PEE-treated C2C12 cells led to a strong induction of VEGF-A in RAW264 macrophages. Moreover, the same supernatant induced MMP-12 and COX2 expression. This induction pattern is reminiscent of angiogenesis caused by tumor-associated macrophages [18]. The healing effects of PEE on muscle might therefore be attributed to angiogenesis caused by the recruited macrophages. Further* in vivo *studies are needed to clarify whether macrophage-elicited angiogenesis occurs in PEE-treated muscles and whether myoblasts are an essential cellular population in that response pathway.

## 5. Conclusions

PEE stimulates myoblasts to secrete the cytokines and chemokines in a NF-*κ*B dependent manner. Subsequently, these humoral factors have the potential to promote macrophage migration and the production of angiogenic factors by macrophages. These results collectively suggest novel beneficial effects of PEE on muscle injury.

## Figures and Tables

**Table 1:** Chemical composition of Brazilian green propolis ethanol extract used in this study.

Propolis	55.0%
p-Coumaric acid	19.3%
Artepillin C	10.3%
Baccharin	3.6%
Drupanin	1.5%
Ethanol	45.0%
